# Percutaneous Coronary Intervention in Familial Hypercholesterolemia Is Understudied

**DOI:** 10.3389/fcvm.2018.00116

**Published:** 2018-08-30

**Authors:** Leo Ungar, David Sanders, Brian Becerra, Ailin Barseghian

**Affiliations:** ^1^Department of Cardiology, University of California, Irvine, Irvine, CA, United States; ^2^Department of Internal Medicine, University of California, Irvine, Irvine, CA, United States

**Keywords:** familial hypercholesterolemia (FH), percutaneous coronary intervention (PCI), statins, PCSK9 inhibitors, revascularization in familial hypercholesterolemia, familial hypercholesterolemia registry, LDL receptor, FH diagnostic criteria

## Abstract

Familial hypercholesterolemia (FH) is a common heritable condition in which mutations of genes governing cholesterol metabolism result in elevated LDL levels and accelerated atherosclerosis. The treatment of FH focuses on lipid lowering drugs to decrease patients' cholesterol levels and reduce their risk of cardiovascular events. Even with optimal medical therapy, some FH patients will develop coronary atherosclerosis, suffer myocardial infarction, and require revascularization. Yet, the revascularization of FH patients has not been widely studied. Here we review FH, identify unanswered questions in the interventional management of FH patients, and explore barriers and opportunities for answering these questions. Further research is needed in this neglected but important topic in interventional cardiology.

## Introduction

Familial hypercholesterolemia (FH) is among the most common heritable cardiovascular conditions, affecting up to 1/250 people worldwide and an estimated 1.3 million people in the United States (1). This genetic abnormality of lipid metabolism is estimated to have an incidence of 1 in 200 to 1 in 500, (2) and has a higher prevalence in certain ethnic groups, including Ashkenazy Jews, French Canadians, Lebanese, and Afrikaners, in which the incidence can be as high as 1 in 67 (3). FH is also burdensome, resulting in accelerated atherosclerosis and increased cardiovascular morbidity and mortality. Yet, it remains widely underdiagnosed, (1) often undertreated, (1) and, with regard to its revascularization management, extremely understudied. As a result, although many patients with FH undergo percutaneous interventions, the optimal approach to these patients is unknown. Here, we briefly review FH (section FH in Review), including its pathophysiology, clinical presentation, diagnostic criteria, and management; identify key unanswered questions related to revascularization in FH (section Interventional Cardiology of Fh: Unanswered Questions), related both to whether and how to intervene; and explore barriers to answering these questions (section Barriers to the Study of Intervention in FH), including a lack of focus on FH patients in existing studies in interventional cardiology, and a lack of focus on intervention in existing studies of FH; and call for further research in this neglected but important area of interventional cardiology.

## FH in review

### Pathophysiology

**Figure 1:**
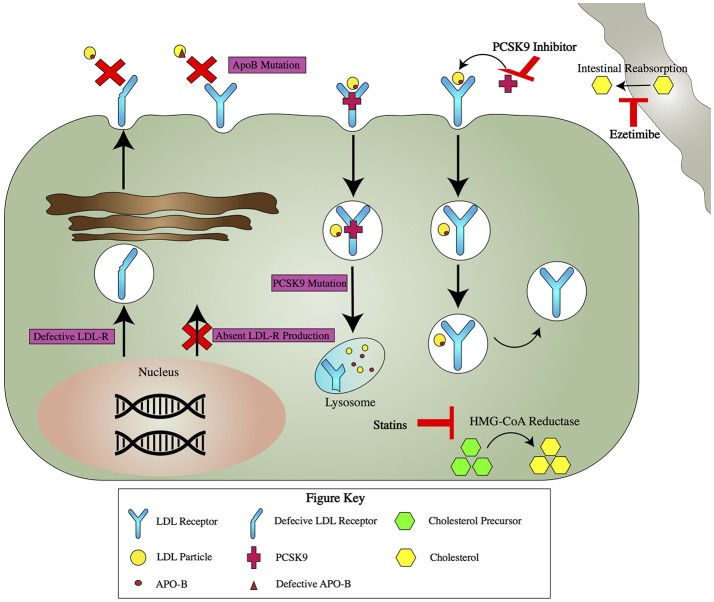
*Pathogenetics of FH* and Mechanism of Action for Key Drug Therapies for FH. *Pathogenetics of FH:* There are four major pathogenetic causes of FH (purple boxes). LDL-R gene mutations can result in production of a defective LDL-R that ineffectively binds LDL or a null allele that leads to no LDL-R production. Mutations in ApoB lead to impaired binding with the LDL-R. PCSK9 mutations lead to increased degradation of the LDL-R. *Key Drug Therapies:* Several drugs act via different mechanisms to lower serum LDL (red T's). Statins inhibit HMG-CoA reductase, the enzyme responsible for the rate limiting step in cholesterol production. Ezetimibe acts away from the hepatocyte, blocking cholesterol absorption in the gut. PCSK9 inhibitors bind PCSK9. This leads to increased recycling of LDL-R and ultimately more available receptors to take up LDL from the serum.

Normally, 70% of LDL is cleared from plasma by LDL receptors on hepatocytes (Figure 1) (2). LDL binds to LDL receptors, which promote the cellular uptake of apolipoprotein B (ApoB) and ApoE containing lipoproteins (2). The LDL receptor, now bound to LDL, is endocytosed into the hepatocyte (4). The LDL receptor is then either recycled to the cell surface or directed by the PCSK9 peptide to degradation by lysosomes (5).

The pathophysiology of FH results from mutations in genes governing cholesterol processing (Figure 1). Three such genes are those coding for LDL receptors, ApoB, or PCSK9 (6). LDL gene mutations are the most common pathogenic mutations in FH, and greater than 1200 LDL gene mutations have been described (7). There are multiple ways to categorize these mutations, but the simplest divides them into two groups, one resulting in a defective LDL receptor, and a second resulting in a null allele which leads to no LDL receptor production. ApoB mutations also cause FH by leading to elevated LDL levels, but by contrast they do this by interfering with the binding of ApoB to the LDL receptor (8). PCSK9 mutations have different mechanisms but all result in increased degradation of the LDL receptor, which results in less LDL endocytosis and higher LDL levels (9). Less common pathogenic FH gene mutations have been described in genes coding for ApoE, LDL receptor adaptor protein, lysosomal acid lipase, and other proteins (10–12).

FH is just one type of heritable condition that results in disordered lipid metabolism. Each of the several disorders is characterized by its specific lipoprotein abnormality and the responsible genetic mutations. FH is the only one that is not associated with abnormal triglycerides. The most common of these heritable dyslipidemias is familial combined hyperlipidemia (FCH), which has an estimated prevalence of between 0.5 and 2% in the general population (13). FCH is a genetically complex disease that can result in significant elevation of both LDL and triglycerides (13). Similar lipid abnormalities, with elevations in LDL and triglycerides, are found in familial dysbetalipoproteinemia, a rare disorder that results from overabundance of ApoE2 protein (14). Both familial hyperchylomicronemia syndrome and primary simple hypertriglyceridemia result in much greater elevation in triglycerides than LDL (15). Familial hyperchylomicronemia, the result of lipoprotein lipase deficiency, is exceedingly rare but leads to severe elevations in triglycerides and pancreatitis (15, 16).

### Clinical features

There are two forms of familial hypercholesterolemia: heterozygous familial hypercholesterolemia (HeFH), in which only one of two alleles in a key gene possesses a pathogenic mutation; and homozygous hypercholesterolemia (HoFH), the rarer genotype, in which both alleles at a locus are mutated.

The clinical consequences of both HeFH and HoFH result from the early and sustained elevation in LDL, leading to premature atherosclerosis and cardiovascular mortality. Children with HeFH are asymptomatic, but their LDL levels may range from 190 to 400 mg/dL (17). Atherosclerosis is accelerated, with subclinical disease detectable by adolescence and premature myocardial infarction possible as early as the third decade of life (18, 19). Without statin treatment, by age 60, the risk of clinically significant CAD may be greater than 50% for men and 30% for women (20). Additionally, patients may have characteristic non-cardiovascular manifestations including tendon xanthomas and corneal arcus (21, 22).

HoFH results in an even more severe phenotype. LDL levels may rise to greater than 500 mg/dL, (23) and atherosclerosis and cardiovascular events can begin as early as the first or second decades of life (24). Atheromatous deposits are extensive, and can involve the carotid arteries, cerebral vasculature, and the root of the aorta, leading to supravalvular aortic stenosis (25). Without treatment, patients with HoFH generally do not survive past 30 years (23).

### Diagnostic criteria

There are three sets of diagnostic criteria for FH: the MED PED, Simon Broome, and Dutch Criteria (Table 1). MED PED is the simplest, using only a patient's age, LDL, and family history. The Simon Broome Criteria also takes into account physical findings and data from genetic testing. The Dutch Criteria includes the most variables and introduces a scoring system in which different features confer a certain number of points, and the likeliness of having FH is determined based on the sum of points. Recently, an app has been developed that in which patients' attributes can be plugged in to determine whether they can be diagnosed with FH by each of these three criteria (26).

**Table 1:** Diagnostic Criteria for FH.

**MEDPED Criteria**				
*Diagnosis based on total cholesterol greater than these values in mg/dL (mmol/L)*				
**Age**	*Relative with familial Hypercholesterolemia*			**General Population**
	**First degree**	**Second Degree**	**Third Degree**	
<20	220 (5.7)	230 (5.9)	240 (6.2)	270 (7.0)
20–29	240 (6.2)	250 (6.5)	260 (6.7)	290 (7.5)
30–39	270 (7.0)	280 (7.2)	290 (7.5)	340 (8.8)
≥40	290 (7.5)	300 (7.8)	310 (8.0)	360 (9.3)
**Simon Broome Criteria**				
**Required for Diagnosis: High cholesterol levels** *Adult:* Total cholesterol >290 mg/dL (7.5 mmol/L) or LDL-C >190 mg/dL (4.9 mmol/L) *Child younger than 16 years old:* Total cholesterol > 260 mg/dL (6.7 mmol/L) or LDL-C > 155 mg/dL (4.0 mmol/L)				
**Definite Familial**		**Probable Familial**		
**Hypercholesterolemia**		**Hypercholesterolemia**		
1. Physical finding of tendon xanthomas, or tendon xanthomas in first or second degree relative *OR* 2. DNA-based evidence of an LDL-receptor mutation, familial defective Apo B-100, or a PCSK9 mutation.		*Family History of:* 1. Myocardial infarction before the age of 60 in a first degree relative or before 50 in a second degree relative *OR* 2. Elevated total cholesterol greater than 290 mg/dL (7.5 mmol/L) in adult first- or second-degree relative or greater than 260 mg/dL (6.7 mmol/L) in child or sibling younger than 16 years old		
**Dutch Criteria**				
**Family History** First degree relative with premature CAD or vascular disease (males <55, females <60 years old) OR LDL-C > 95th percentile for age and gender				1
Tendon xanthomata and/or arcus cornealis				2
First degree relative <18 years old with LDL-C>95th percentile for age/ gender				2
**Clinical History** Patient with premature CAD				2
Patient with premature peripheral vascular and/or cerebrovascular disease				1
**Physical Exam** Tendon xanthomata				6
Arcus cornealis prior to age 45	4
**Cholesterol Level:** LDL-C mg/dL (mmol/liter) ≥ 330 (≥8.5)				8
250–329 (6.5–8.4)				5
190–249 (5.0–6.4)				3
155–189 (4.0–4.9)				1
**DNA** Functional mutation in LDLR, Apo B, or PCSK9 gene				8
**Diagnosis** Definite Familial Hypercholesterolemia				≥8
Probable Familial Hypercholesterolemia				6–8
Possible Familial Hypercholesterolemia				3–5
Unlikely Familial Hypercholesterolemia				<3

### Management

Currently, the management of FH focuses almost exclusively on the use of medications to lower the LDL (Figure 1). First line therapy is a high intensity statin, with a goal of reducing LDL by >50% (27). If this goal is not achieved with statin therapy alone, ezetimibe can be added (28, 29).

If a >50% reduction in LDL is achieved but LDL remains above 100 in patients with no history of clinically significant atherosclerosis, or above 70 in patients with a history of atherosclerosis, it is recommended to add ezetimibe if <25% additional LDL lowering is required, or a proprotein convertase subtilisin/kexin type 9 (PCSK9) monoclonal antibody if >25% LDL reduction is needed (30). PCSK9 normally binds to LDL receptors and causes their degradation. PCSK9 inhibitors, such as alirocumab and evolocumab, work by inhibiting PCSK9 and decreasing LDL receptor degradation, which results in an increased number of LDL receptors, which in turn results in increased resorption of LDL from the bloodstream and lower LDL levels (31, 32).

Non-medication therapies include lipophoresis, which is beneficial in HoFH, (33) and, as a last resort, liver transplant, which has also been used in HoFH including preemptively before vascular complications arise (34, 35).

Novel therapeutics are in development. Mipomersen works by the novel mechanism mRNA inhibition, (36) binding to the mRNA of Apo B-100, resulting in mRNA degradation by ribonuclease H1 and decreased Apo B-100 production. Apo B-100 is an integral part of the formation of VLDL, and decreasing Apo B-100's production results in a lowering of LDL (36). Another novel therapeutic agent, lomitapide, also works via inhibition of VLDL formation. Lomitapide prevents VLDL production by inhibiting microsomal triglyceride transfer protein, which is essential in the assembly of VLDL by the liver (37). Both mipomersen and lomitapide have been FDA approved for the treatment of HoFH and are currently under investigation for use in HeFH (38, 39) A third agent that is still under investigation is inclisiran, a small interfering RNA (siRNA) that inhibits PCSK9 production by selectively silencing the translation of the messenger RNAs for PCSK9 (40–42).

LDL lowering in FH reduces atherosclerosis and improves outcomes. In FH patients on lipid lowering therapy, LDL levels have been correlated with coronary artery disease severity by CT coronary angiography (43). Lipid lowering by medical therapy combined with lipophoresis has been associated with plaque regression as assessed by coronary angiography and coronary intravascular ultrasound (44). Lipid lowering also reduces cardiovascular adverse events, and if goal LDL levels are reached, FH patients can achieve a risk of MI similar to that of the general population (1, 45, 46).

The goal of medical therapy is primary or secondary prevention, to reduce the risk of a patient having either a first or second atherosclerotic event (47, 48). Even with optimal therapy, however, some patients will develop coronary artery disease, suffer myocardial infarction, and require revascularization. Yet, the interventional care of FH patients remains understudied. We identify key questions facing the interventional cardiologist caring for FH patients, discuss some of the challenges in answering these questions, and call for further research.

## Interventional cardiology of FH: unanswered questions

### When to intervene

The initial question facing interventionalists, for patients with and without FH alike, is always whether and when to intervene (Table 2). In STEMI(49, 50) and NSTE-ACS(51) primary percutaneous intervention is not controversial, with multiple studies demonstrating the efficacy of primary PCI in those settings. Nor should it be controversial in STEMI and NSTE-ACS patients with FH. In other settings, however, the benefits of percutaneous intervention are less clear, rendering it ambiguous whether intervention should be pursued.

**Table 2:** Revascularization in FH: Unanswered Questions, Barriers to Study, and Future Directions.

**Unanswered Questions 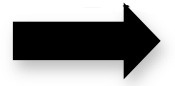 **	**Barriers to the Study of Revascularization in FH**	** 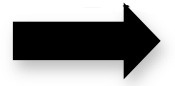 Going Forward**
– **When to Intervene?** • Stable Angina • Angiography and FFR – **How to Intervene?** • PCI vs. CABG • Potential bioabsorbable stents	– **Revascularization studies have not focused on FH**. • *FH patients have not been pre-identified in studies* – **FH studies have not focused on revascularization** • *FH studies have focused on medical therapy, not revascularization*	– Bioabsorbable stents a potential use in children and younger patients with FH? – Genetic testing to identify FH patients in studies, despite increased cost? – Using H&P to identify FH patients according to the MED PED/Dutch/Simon-Broome criteria? – Focused prospective studies to include revascularization when collecting data?

#### Stable angina

There is a longstanding and ongoing debate about the benefits of revascularization in stable angina. The COURAGE Trial demonstrated that angiography-guided PCI in stable angina was not superior to optimal medical therapy (OMT) (52). Conversely, FAME-2 showed FFR-guided PCI in stable angina was superior to OMT with regard to a primary composite endpoint that included all-cause mortality, non-fatal MI, or unplanned hospitalization with urgent revascularization (53). However, the only one of the three individual components of this composite outcome that PCI significantly reduced was urgent revascularization, causing some to argue against even FFR-guided PCI for stable angina on the grounds that it is tantamount just to performing a PCI to prevent a PCI (54). PCI is also often thought to reduce symptoms in stable angina, but that was called into question more recently in the ORBITA trial, which randomized patients with stable angina to PCI vs. a sham procedure stimulating PCI, and which demonstrated no benefit of PCI relative to the sham procedure with regard to symptoms or function (55).

The issue here is whether these data are generalizable to FH. None of these studies were limited to a FH population, or resulted in secondary analyses examining an FH subpopulation. The generalizability of these data to FH patients is therefore unclear. It may be that FH patients have more (or less) benefit from revascularization in stable angina, and further studies of FH patients specifically would be required to clarify this issue.

#### Angiography and FFR

A second consideration is angiographic and FFR- or iFR-guided PCI. The question here is how are outcomes impacted by not intervening on lesions that are angiographically intermediate but not physiological significant by FFR or iFR? In non-FH patients, the FAME trials demonstrated that FFR-guided PCI is at least on par with angiographic-guided PCI (non-inferior with regard to certain outcomes, superior with regard to others), (53, 56, 57) and more recently DEFINE-FLAIR (58) and iFR-SWEDEHEART (59) showed outcomes with iFR are equivalent to those with FFR, arguing that iFR guidance, too, is likely superior to angiography alone, even after five years of follow-up (60). These trials did not have subanalyses of an FH population, however, so their implications for FH patients are unclear. Theoretically, one might imagine that, given that FH patients often get clinically significant atherosclerosis younger than do non-FH patients,(18, 19) plaques could progress more quickly in FH patients than in non-FH patients, especially if FH patients are statin non-adherent and their LDL remains high. That hypothesis, if true, could provide reason to question whether hemodynamic biomarkers of plaque significance provide the same reassurance for long-term follow-up in FH, both for prognosis and for treatment effect (60).

### How to intervene

The second question facing interventionists is, once it has been decided that revascularization should be performed, how should it performed? Two considerations we address here include PCI vs. CABG, and whether bioabsorbable stents may have a unique role to play in intervention in very young HoFH patients.

#### PCI vs. CABG

In non-FH patients, PCI vs. CABG has been studied extensively (61–65). Essentially, differences between outcomes for PCI and CABG have not been demonstrated, except in a few key situations in which CABG has been associated with improved outcomes as compared to PCI, including: patients with diabetes and 3-vessel disease, and patients with or without diabetes with 3-vessel disease and intermediate or high SYNTAX scores (66, 67). A theory is that in patients with more severe disease, the benefit of CABG results from bypassing not only the lesions that are culprit at the time of intervention, but other lesions that may develop in the future.

This logic could be applied to FH. FH patients, just like diabetics and patients with multivessel disease, may be more likely to have future lesions, and so CABG may have additional benefit in bypassing the lesions of both today and tomorrow. On the other hand, against CABG for FH patients may be that they are often younger at the time of their MI, and venous grafts are known to close off, at a rate of approximately 50% graft failure by 10–15 years, (68–74) potentially making them less viable for younger, otherwise healthy patients who may require their revascularization to be longer-lasting. These theoretical considerations should be viewed as hypothesis generating, and studies should be performed comparing PCI to CABG in FH patients.

#### Potential of bioabsorbable stents

The second consideration is, if PCI is performed, what sort of stent should be inserted, especially in HoFH patients who can have MIs even as young children? In general, drug-eluting stents (DES) have become the stent type of choice, as landmark trials demonstrated that they have decreased rates of in-stent thrombosis and restenosis as compared to bare metal stents (75, 76). These stents remain in the coronaries permanently, which is usually not an issue, but a theoretical concern, perhaps unique to HoFH, is that even young children can have myocardial infarctions and require revascularization, and stents that are appropriately sized for child-sized coronaries may not remain so after the child, and their coronaries, grow.

A new technology may help with this issue. Bioabsorbable stents are made of a special polymer which, over time, dissolves completely, leaving no residuum in the artery in which it was implanted (77). This technology initially generated excitement and investment, and gained FDA-approval in 2016 (78). After further studies demonstrated lower efficacy and higher thrombotic rates with bioabsorbables, (79) however, in 2017 they were pulled from the market by their manufacturer. One very specific area in which they may have special utility is in percutaneous intervention in children. Notably, a slew of case studies have been reported in which PCI using a bioabsorbable stent was performed on a child with FH, with the hope that its unique capacity to entirely dissolve would yield special benefit to the pediatric patient whose coronary arteries are still growing by circumventing an eventual size mismatch between stent and artery (80, 81). This creative idea thus far is the topic of case reports only, but more longitudinal study of these patients could reveal whether there is any actual evidence of this hypothetical, if nevertheless plausible, advantage.

## Barriers to the study of intervention in FH

As noted above, data on the interventional cardiology of FH are lacking. Here we propose two reasons why this might be as well as two possible opportunities going forward (Table 2).

### Interventional studies have not focused on FH

First, most of the studies that have been performed previously do not have the granularity to identify FH patients. Trials generally assess their study drug's impact on particular patient populations by preidentifying specific patient populations in advance of the trial (such as diabetic patients), and then making sure to identify which patients in the study overall have the condition of interest. Regrettably, many clinical trials do not collect the data that would allow investigators to identify which study-patients have FH, specifically family history, key physical exam findings (tendon xanthoma and arcus cornealis), and genetic testing. We suggest that future trials include FH patients among their preselected subpopulations for further study, and collect the data necessary to identify them.

A possible barrier to this approach is that performing genetic analysis is costly, ranging from $620 to 1,485 according to one report, (82) and doing so for all trial participants would likely be cost-prohibitive. A possible workaround would be to collect all the relevant data besides the genetics (specifically family history and physical exam findings of tendon xanthoma and arcus cornealis) and proceed as best as possible with the available information, which could still yield diagnoses, by Dutch and/or Simon-Broome Criteria, of possible or probable FH in many cases (3). The optimal way to design trials to identify FH patients with high sensitivity and specificity while avoiding prohibitive cost increases merits further consideration.

### FH studies have not focused on percutaneous intervention

Second, the studies that do focus specifically on the FH subpopulation have focused more on medical therapy and neglected to look at outcomes after percutaneous intervention. The PCSK9 trials, to their credit, examined outcomes in FH patients, but these obviously investigate a medical rather than an interventional therapy (83). The CASCADE FH Registry is a promising, longitudinal, observational study which as of 1/8/2017 had enrolled 3,960 patients across the US (84). Despite the CASCADE FH registry yielding multiple reports on diverse topics in FH, (85–87) to our knowledge, it has not yet reported on outcomes after intervention, and exactly what data it is collecting on interventions in FH patients is unclear. Neither have any other FH registries, to our knowledge, reported analyses of outcomes with interventional therapies, specifically. We suggest that these focused prospective studies of the FH patients, both randomized studies of particular therapies and observational registries, collect data on interventions and report outcomes after FH patients have undergone intervention.

## Conclusion

FH is a genetic disease of lipid metabolism that is common and burdensome but underdiagnosed and undertreated (1). Current management is primarily medical, includes therapeutics that are both established (statins, ezetimibe) and novel (PCSK9 inhibitors, mipomersen, lomitapide), and focuses on lipid lowering for primary and secondary prevention of cardiovascular events. Revascularization in FH, however, is extremely understudied. As a result, although many patients with FH undergo percutaneous coronary interventions, the optimal interventional approach to these patients is unknown. Key unanswered questions relate to both whether (In stable angina?) and how (CABG vs. PCI? Bioabsorbable stents?) to intervene. Barriers to answering these questions include a lack of focus on FH patients in existing studies in interventional cardiology, and insufficient concentration on percutaneous intervention in existing studies of FH. Further research on coronary revascularization in FH is needed to guide the interventional management of FH patients.

## Author contributions

All authors listed have made a substantial, direct and intellectual contribution to the work, and approved it for publication.

### Conflict of interest statement

The authors declare that the research was conducted in the absence of any commercial or financial relationships that could be construed as a potential conflict of interest.
